# An Adaptive, Situation-Based Risk Assessment and Security Enforcement Framework for the Maritime Sector

**DOI:** 10.3390/s22010238

**Published:** 2021-12-29

**Authors:** Christos Grigoriadis, Romain Laborde, Antonin Verdier, Panayiotis Kotzanikolaou

**Affiliations:** 1 SecLab, Department of Informatics, University of Piraeus, Karaoli & Dimitriou 80, 18534 Piraeus, Greece; 2 Institut de Recherche en Informatique de Toulouse (IRIT), Université Paul Sabatier, 31062 Toulouse, France; Romain.Laborde@irit.fr (R.L.); antonin.verdier1@univ-tlse3.fr (A.V.)

**Keywords:** adaptive security, event management and analytics, situation-based risk assessment, situational policy elicitation and enforcement

## Abstract

Maritime processes involve actors and systems that continuously change their underlying environment, location and threat exposure. Thus, risk mitigation requires a dynamic risk assessment process, coupled with an adaptive, event driven security enforcement mechanism, to efficiently deal with dynamically evolving risks in a cost efficient manner. In this paper, we propose an adaptive security framework that covers both situational risk assessment and situational driven security policy deployment. We extend MITIGATE, a maritime-specific risk assessment methodology, to capture situations in the risk assessment process and thus produce fine-grained and situation-specific, dynamic risk estimations. Then, we integrate DynSMAUG, a situation-driven security management system, to enforce adaptive security policies that dynamically implement security controls specific to each situation. To validate the proposed framework, we test it based on maritime cargo transfer service. We utilize various maritime specific and generic systems employed during cargo transfer, to produce dynamic risks for various situations. Our results show that the proposed framework can effectively assess dynamic risks per situation and automate the enforcement of adaptive security controls per situation. This is an important improvement in contrast to static and situation-agnostic risk assessment frameworks, where security controls always default to worst-case risks, with a consequent impact on the cost and the applicability of proper security controls.

## 1. Introduction

**Figure 1:**
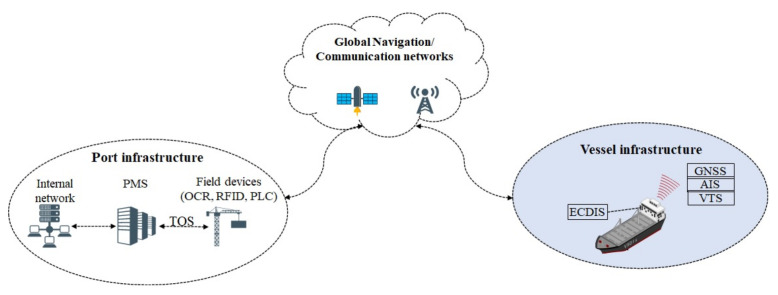
An overview of maritime information and communication systems.

Maritime transport is a complex environment involving various actors with different objectives, cyber and physical components and interconnected systems. It utilizes critical infrastructures and systems for service provisioning, such as port facilities and specialized systems. In modern maritime systems, most processes are (semi)automated and controlled by maritime SCADA, which control the underlying systems. At the port side, the Port Management System (PMS) orchestrates all the supply chain processes, by receiving information from the Terminal Operating System (TOS), which monitors the location of containers and handling of other equipment (e.g., cranes) through Optical Character Recognition (OCR), Radio Frequency Identification Devices (RFIDs) and GPS systems (see Figure 1). Typical ship side systems include: the Automatic Identification System (AIS), a tracking system transmitting information related with the course, speed or type of cargo, which is mainly used for collision avoidance; the Vessel Traffic Service (VTS), which is mainly for marine traffic monitoring; and the Electronic Chart Display Information System (ECDIS), a navigational chart display that receives data by other control systems, to assist ship crew in ship navigation.

Typical types of maritime communications involve *Ship-to-Port* (e.g., Maritime Single Window (MSW) reporting, including ship certificates, notifications or requests); *Ship-to-Ship* (e.g., broadcast AIS info to nearby ships for collision avoidance); and *Port-to-Ship* communications (e.g., broadcast of available channels, GPS corrections or weather reports).

*Cybersecurity challenges in maritime sector.* The increased interconnection and connectivity of maritime systems has created a new threat landscape and new security challenges in the maritime sector, such as man-in-the-middle and hijacking attacks on AIS systems against nearby ships, spoof ship positioning systems or even deviating the ship course by broadcasting modified GPS signals [1]. Recent security incidents also include ransomware attacks against maritime systems such as the NotPetya attack [2], or hybrid cyber-physical attacks by tech-savvy pirates that will first breach the shipping company servers to locate the most profitable target vessel, based on its cargo, before physically attacking to the vessel [3].

**Figure 2:**
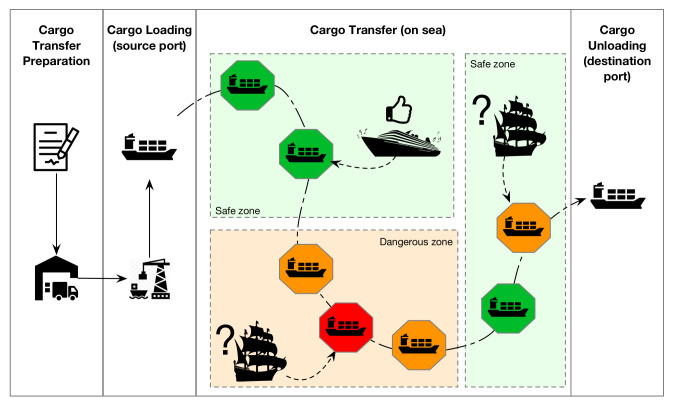
Maritime scenario: Security situations of the vessel.

*The need for situational awareness in maritime security: A motivating example.* Besides the sharp increase in cybersecurity threats, maritime transport is an inherently agile environment, in terms of environmental changes, applicable threat agents and attack vectors. We will utilize a typical maritime transport service, mainly *cargo transfer*, to demonstrate how environment changes affect the underlying risks of systems and consequently the need for an adaptive security framework. Cargo transfer is usually initiated by a third party, a merchant who sends a purchase order to the producer. After the contract terms (e.g., pricing, documentation, freight charges) have been agreed on, the producer contracts a ship agent to deliver the cargo to the destination port. The ship agent makes the arrangements with the ship owner to assure usage of ships; with customs authorities to arrange for the manifest registration number; with the departure port authority to arrange the ship formalities related to the authorization process from the entry of the ship into the port until its exit and then proceed to load the cargo into the vessel for shipment to the destination port. The ship agent contracts a cargo transport agent and assigns the transfer of cargo from the industry to the departure port. Finally, the ship agent sends the relevant documentation to the importer’s local agent who has the responsibility for the ship arrival and the regional procedure of delivering the vessel to the importer (see Figure 2).

Obviously, during the cargo transfer a ship may experience various environmental changes, e.g., route through safe and non-safe sea areas, be in proximity with known, unknown or even hostile vessels, experience variations in network connectivity, etc. Therefore, as the situational changes affect the applicable threats, threat agents and effective attack vectors, they consequently affect the resulting security risks in a dynamic manner.

*Existing solutions.* Over the last years several maritime specific risk assessment methods have been proposed in the literature, e.g., [4,5,6], to capture the specific characteristics of the maritime threat environment as well as the security needs of the maritime supply chains. However, all existing maritime RA methodologies are *static*, in the sense that they do not support dynamic changes in the risk assessment, to adaptively capture variations in the risk level derived from situational changes and/or other events. For example, in the cargo transfer scenario described above, a cargo vessel entering a ‘dangerous sea area’ (situational change) may experience a higher risk related with physical threats. In addition, when an event such as the ‘proximity with an unknown vessel’ is detected, security risks related with the integrity of the communication systems should be re-estimated. Indeed as recent real-world incidents have demonstrated (https://www.gpsworld.com/spoofing-in-the-black-sea-what-really-happened/ (accessed on 15 November 2021)) nearby attackers may successfully jam, intercept or inject fake messages against improperly secured sea communication or navigation systems.

A simple solution, followed by existing methodologies is to default to the worst-case scenario by applying the ‘strongest’ security controls, in order to assure the highest level of authenticity, integrity, availability, confidentiality, non-repudiation and resilience at all situations. Although this policy seems as “being on the safe side”, it affects the operation cost and consequently the actual enforcement of security controls. Applying the strongest security controls is not always possible in maritime systems due to environmental constraints. For example, limitations in network connectivity may prevent the continuous application of security controls that require online verification. In addition, as maritime is a sector with low profit margins, the administrators usually phase limitations in resources for cybersecurity investments. Thus the security controls should continuously adapt to environmental changes. Risk assessment and mitigation should take into consideration the situational changes and the environmental constraints, in order to dynamically apply those security controls that can continuously maintain the situational risk bellow the risk threshold, but in a cost-efficient manner.

*Contribution.* In this paper, we propose an adaptive security framework that covers both dynamic risk assessment and situational driven security policy deployment. We extend a maritime-specific risk assessment methodology (MITIGATE [5,6]) to suggest adaptive security controls, and integrate it with a situation-driven security management framework (DynSMAUG [7,8,9]) to dynamically enforce adaptive security policies implementing the security controls. We will follow a situation-driven approach. Situations allow capturing complex and dynamic constraints (e.g., time, location, workflows, etc.) Moreover, situational awareness is about understanding the context and being able to project in the future to improve decision making. This concept will provide a guide for maritime security assessment and security policy enforcement. The resulting framework will be able to suggest adaptive security controls for various critical functions of the cargo transport service required by each security level of each situation. The situations identified at the risk assessment stage will be formally specified using complex event techniques while situation-driven security control will be translated into situation-driven security policies. The underlying security management infrastructure will then be able to dynamically deploy and enforce security policies making security adaptable to each predefined situations and security levels.

*Paper Structure.* In Section 2, we review the related work. In Section 3, we propose an adaptive security framework for dynamic risk assessment and situational driven security policy deployment. In Section 4, we validate our methodology, by applying it in a realistic scenario, for maritime cargo transfer service. Section 5 discusses the advantages and limitations and concludes this paper.

## 2. Related Work

### 2.1. Risk Assessment and Cybersecurity in the Maritime Sector

Despite the need for adaptive risk management in the area of maritime sector, currently very few approaches support, to some extent, real-time and ‘live’ risk assessments. These include MEDUSA [4], MITIGATE [5,6] and TREsPASS [10], which partially automate the risk assessment process, by enabling users and stakeholders to collaboratively and asynchronously provide their input for conducting dynamic reassessments. Although some automation is supported, they cannot be considered as autonomous risk assessment systems.

The lack of adaptive risk assessment methodologies is not only related with maritime transport, but is common in other sector specific methodologies with a very few exceptions. For example, in [11] RiskMon, an automated RA framework is proposed aiming to reassessing the risk for mobile phone applications. RiskMon assigns a risk score on every access attempt on sensitive information and ranks applications by their cumulative risk scores. In [12], a game-theoretic model for automated risk mitigation in cloud systems is proposed. Its goal is to model the cost-benefit analysis of alternative mitigation controls in real-time. The main motivation is to reduce the cost of applying security controls that may offer a very high, but unnecessary security level with respect to the current security risks, by providing a trade-off between the effectiveness of the adaptive risk treatment and the cost resulting from the execution of the selected security mechanisms. The objective is to implement dynamic security policies that adapt to the dynamic nature of the cloud in a nearly cost optimal way.

Adaptive security approaches are quite needed in the maritime environment as well, to assure effective and cost efficient cyber threat preparedness. This issue is discussed in [13], which identifies the need to attach cybersecurity related matters and cyber threat prevention more systematically in the maritime logistics industry, while also considering these aspects when new technologies are being implemented. In [14], reasons for raising awareness on this issue of cybersecurity in ports and generally the maritime industry are pointed out. It is derived that the development of methodologies to objectively assess cyber risks and mitigate their effects in the port industry should be further investigated, especially in the era of IoT. In an attempt to manage the information flows throughout the maritime infrastructure, in [15], a dynamic security visualization platform for operational maritime cybersecurity is presented. The platform can flexibly support collaboration among multiple stakeholders for asset modeling, while introducing multiple security roles to increase situational awareness. This map of interconnections introduces further layers of information to current risk assessment procedures and raises multiple questions toward current risk models.

Having in mind the broader infrastructure of the maritime supply chains, the various assets involved and their related challenges and threats must be identified. In [16], by analyzing available resources, a shift in mind-set is proved as essential to direct more attention and resources toward cybersecurity. The need for multiple positioning, navigation, and timing (PNT) systems onboard maritime vessels to complement GPS-only navigation is identified. To further point out this need, in [17], simulated attacks towards current navigation systems are implemented. The feasibility of an attacker using a radar system or AIS as an open door to remotely send commands to a cyber threat hosted on a ship is investigated. A triggering mechanism that uses a template matching technique to detect specific patterns transmitted by the attacker to the ship’s radar or AIS is proposed. Finally, in [18], major security weaknesses affecting systems and communication technologies adopted in modern vessels are researched, paying specific focus to the architecture and the main features of various naval systems like GNSS, AIS and satellite communications. Exploitation vectors and the corresponding countermeasures are identified for the systems. Such approaches should be considered throughout the risk assessment procedures, while enumerating threats and vulnerabilities.

To provide optimization on security issues in a higher level, in [19], an approach to identify cybersecurity risk components in the maritime sector and to derive priorities for vulnerability improvement plans through itemized risk assessment is presented. To this end, qualitative risk assessment (RA) was carried out for administrative, technical, and physical security risk components based on industry and international standards, which were additionally presented in the International Maritime Organization (IMO) guidelines. Additionally, in [20], an integrated method for safety and security requirements engineering for cyber physical systems at the design stage of the system lifecycle is proposed. This method identifies security and safety objectives, and systematically elicits a comprehensive list of requirements, which are then linked to objectives. Such approaches contribute a lot to risk assessment procedures applied to maritime systems, as they set a standard for modeling risk and security requirements.

In [21], which assesses novel security models, metrics and security assessment for maritime vessels, the lack of capabilities to efficiently manage the identification of vulnerabilities, security risk assessment, and evaluate the effectiveness of countermeasures is considered. To resolve this issue, a novel framework and security risk modeling and assessment method to evaluate the security of maritime vessel networks is proposed. This framework contains a security model able to capture vessel systems, events, vulnerabilities and network configurations and connectivity. Another approach that presents a novel cyber-risk assessment method for ship systems can be found in [22], in this case, the Cyber-Preliminary Hazard Analysis method steps are enriched with new steps supporting the identification of cyber-attack scenarios and the risk assessment implementation. The proposed method is applied for the cyber-risk assessment and design enhancement of the navigation and propulsion systems of an inland waterways autonomous vessel. Finally a dynamic and adaptive security policy enforcement framework for ships is presented in [23]. The CyberShip-IoT framework is proposed to provide a network level defense for the communication network component of ship systems, which offers a high-level policy language and a translation mechanism for automated policy enforcement in the ship’s communication network. Approaches like that can feed information to adaptive risk assessment procedures.

### 2.2. Situations and Situational Awareness

Although the word *situation* is commonly employed in ordinary, legal or even technical domains, many definitions have been published [24]. Especially, *situation* is often interchanged with the word *context*. This section presents the definitions of context and situation that will be used in this article.

Dey [25] has proposed one of the most popular definitions of *context* in the context-aware computing community: “*context is any information that can be used to characterize the situation of an entity. An entity is a person, place, or object that is considered relevant to the interaction between a user and an application, including the user and applications themselves*”. Hence, *situation* is something more abstract than *context*.

Endsley [26] supplemented this work in her definition of situational awareness, which is *the perception of the elements in the environment within a volume of time and space, the comprehension of their meaning, and the projection of their status in the near future.* This definition stresses different characteristics of situation. Situation awareness includes making relations between context information, understanding context and being able to project in the future. As a consequence, a situation can be qualified as “secure” or “unsecure” unlike context.

Finally, psychology has also proposed to define situation models for describing the process of language comprehension and memory. Zwaan and Radvansky [27] consider five dimensions of situations: *time, space, causation, intentionality, and protagonists/objects* where dimension ‘protagonists/objects’ is the ‘meat’ of the situation models, dimension ‘intentionality’ refers to the goals of protagonists and dimension ‘causation’ deals with evidences inferred by events.

## 3. The Proposed Methodology

**Figure 3:**
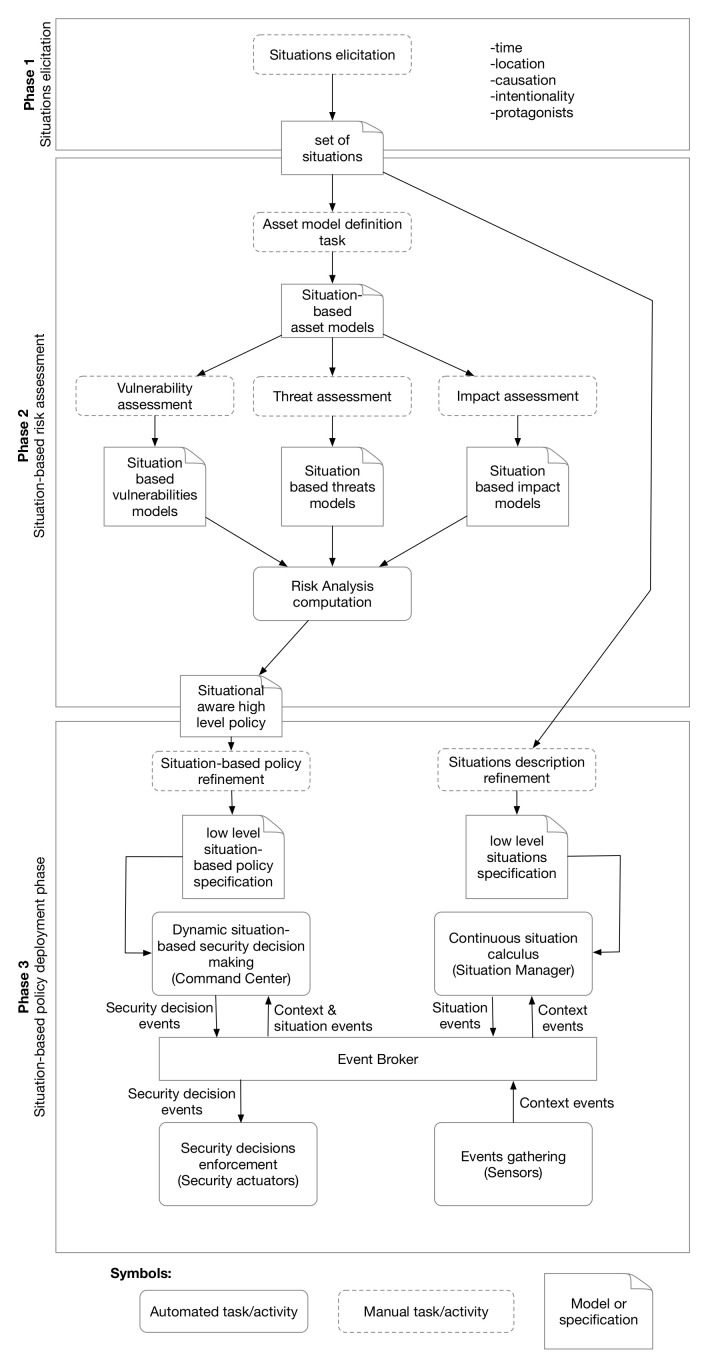
The proposed methodology.

We extend and combine the MITIGATE maritime risk assessment methodology, with the DynSMAUG situation-driven security management framework, to dynamically enforce adaptive security policies. The proposed methodology, illustrated in Figure 3 is comprised of three phases, described in detail bellow: (1) situations elicitation, (2) situation-based risk management and (3) situation-based policy deployment. Note that although the methodology is specifically crafted for the maritime sector, it is possible to extend it for other critical sectors, by properly adjusting the situation elicitation and other tasks, such as the threat agent mapping, to other sectors.

### 3.1. Phase 1: Situations Elicitation

The first phase focuses on eliciting the set of situations in which a vessel can be found. The purpose is to unveil potential cyber, physical and cyber-physical attack paths, throughout the infrastructure used in the context of the cargo transport service. Going further to specify a situation, we study which threat agent profiles have the required capabilities to exploit each identified attack path, and which are the security policy shortcomings that may allow such an event in a specific time and place:

- Where is the vessel located at this point in time?
- Which vessel systems should be active in this specific location? (Hardware/Software)
- Which vessel communication channels should be active in this specific location? (related to threat actors, interference from natural phenomena and equipment restrictions)
- What does the security policy dictate for human-to-equipment interaction and equipment-to-equipment interaction in each specific location?
- Which external threat actors are most active and which internal threat actors are likely to have sufficient access to initiate an attack towards critical assets in this location?

The purpose and significance of considering situations for maritime cybersecurity, are highly related to the complex nature of the underlying environment. To design a situational maritime security approach, we will take into consideration an abundance of parameters that can be boiled down to the five dimensions of the situation model proposed by Zwaan and Radvansky [27]:

- The *Protagonists/objects dimension* in the context of maritime transport security requires the study of human and system agents that can potentially interact with the vessel. This includes internal and external actors, such as human or system agents acting on ports, vessels or elsewhere. At the same time, actors may be trusted (e.g., a port official adhering to the protocol) or malicious (e.g., a disgruntled employee or pirates at open seas)/actors may include not only humans be systems as well. Active systems/assets and cataloged information also pertains to this dimension.
- The *Space dimension* relates to the evolution of the physical locations of the vessel and other protagonists.
- The *Time dimension* includes topics related to time periods, maritime transport workflow steps regarding the mission of the vessel, etc.
- The *Causation dimension* deals with deducing evidences that can be inferred by other contextual data. For instance, analyzing the speed and trajectory of another vessel may reveal that both vessels will be in physical proximity in the near future.
- The *Intentionality dimension* focuses on the goals of the protagonists. Attackers have threat goals while honest parties perform tasks that adhere to their role in the system. As a consequence, security policies and procedures that dictate the behavior of human are studied in this dimension too.

**Figure 4:**
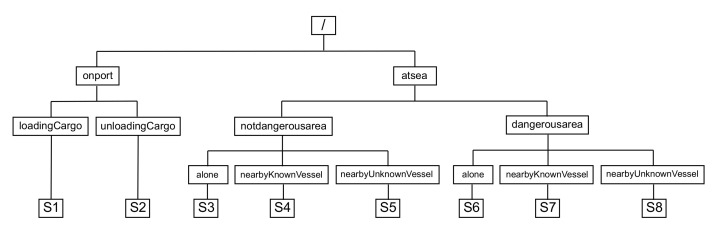
Maritime scenario: Decision tree based situation elicitation.

We propose to organize the elicitation of situations by combining the five dimensions of situations and a situation tree structure. A situation tree is a mind map where each level of the tree corresponds to a specific question dedicated to one dimension (e.g., the question ’Where is the vessel?’ refers to the space dimension). Sibling nodes in a situation tree are literals representing the possible answers for the question (e.g., the vessel can be *on port* or *at sea*). The leaf nodes are the situation names. The definition of each situation is the path from the root node to the leaf. The resulting situation clause is the conjunction of node literals in the path. By applying our situation elicitation methodology on the cargo transfer service, a concrete set of situations is produced, as shown in Figure 4. For instance, situation S1 means the vessel is on port and loading cargo, while situation S8 corresponds to the vessel is at sea and in a dangerous area and nearby an unknown vessel.

### 3.2. Phase 2: Situation-Based Risk Assessment

**Figure 5:**
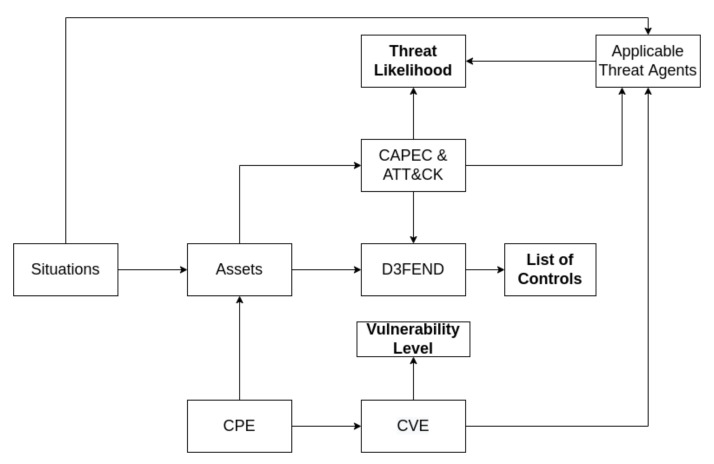
Relations among risk related entities and relevant datasets.

Based on the situations defined in the previous phase, all the risk assessment tasks defined in the MITIGATE methodology such as asset modeling, threat, vulnerability and impact assessment are properly adjusted to each situation, to output a fine-grained, situational risk assessment. As defined in [5,6], MITIGATE is a maritime specific risk assessment methodology, built in compliance with international security and risk management standards such as ISO 27001 [28], ISO 27005 [29] and NIST SP800-30 [30]. In MITIGATE assets, threats, vulnerabilities and threat agents are instantiated with the use of datasets pulled from open sources provided by widely known organizations like MITRE and the National Institute of Standards and Technology (NIST). In particular, for each asset, we identify relevant vulnerabilities based on the Common Vulnerabilities and Exposures (CVE (https://cve.mitre.org/ (accessed on 1 September 2021)) database. As illustrated in Figure 5, threats are instantiated based on the Common Attack Pattern Enumeration and Classification (CAPEC (https://capec.mitre.org/ (accessed on 1 September 2021))) catalog and the Adversary Tactics Techniques and Common Knowledge (ATT&CK (https://attack.mitre.org (accessed on 1 September 2021))) framework, while security controls are D3FEND (https://d3fend.mitre.org/ (accessed on 1 September 2021)) matrix.

In addition, new relationships are created by utilizing common characteristics that are utilized in the above datasets. For example, going through the CAPEC catalog, we identified attributes related with threat agent profiles, such as the *resources*, the *skills* and (iii) the *motivation* (consequences for CAPEC). Therefore, we can identify the threat agents that have the required skills, resources and motivation to take advantage of a specific threat that resides in a specific asset. Finally, for each situation, it is possible to directly map different instances of the active assets and threat agents, while we can also map indirectly different threat and vulnerability levels by using the common characteristics of the underlying datasets.

#### 3.2.1. Situational Asset Model Definition

Situation elicitation is used as an input to define the situation-based risk assessment phase. Based on the defined situations a manual asset modeling process is used to defined alternative asset models that represent the different situations. In contrast to risk assessment approaches that do not define situations, e.g., [4,5,6], in our methodology, for each situation, different asset models are defined to capture the interconnections and the dependencies among assets in different situations.

##### Step 1—Service Identification

The first step involves the identification of the available internal maritime services for an organization. A comprehensive list of all maritime services along with their corresponding processes must be generated, such as cargo loading, cargo transfer, cargo unloading, etc. A service is a collection of processes that are part of a specific maritime ecosystem and may depend on external actors. The dependencies between services and business partners, as well as services and processes must be identified, so that the risk assessment can proceed.

##### Step 2—Asset Identification and Cataloguing

Having identified the available services and processes, the next step is to decompose each process, identify the assets on which it depends on and define the asset criticality. These assets would mainly be internal system components that are controlled by the examined organization(s). The available asset types in the context of our methodology are hardware, and software assets, where the latter are divided to operating systems and application software. The specific entries are derived from the Common Platform Enumeration (CPE) catalog. While the underlying MITIGATE methodology defines assets without considering different situations, in the proposed methodology asset identification is specific to each defined situation. A different asset map is performed and cataloged for each situation, in the context of the identified maritime processes. For example, vessels utilize various communication and navigation systems such as MSW, ship reporting systems (SRS) and Automatic Identification Systems AIS only when the ship is on route, while the same systems are inactive while the ship is in the port. In the same way, collision avoidance systems may be activated on route or may be in a different state before the departure or upon arrival. Hence, a different asset map applies to different situations, and assets have a different criticality level for different services and situations, defined in a simple [High,Medium,Low] scale.

#### 3.2.2. Situational Threat Assessment

Having completed the situation-based asset models defined in the previous phase, the threat assessment procedure can be implemented utilizing the recorded information. While in MITIGATE [5], threats are defined for each asset and are situation-agnostic, in the proposed methodology, we define applicable threat agents that may activate specific threats per situation. This involves the following steps:

##### Step 1—Threat Mapping

For each situation, the involved assets and the applicable threats are mapped. Due to functionality, security and costs, all assets might not be active in all situations. Furthermore, some threats may be easy to activate in some situations only. For example GPS spoofing is an active threat when a vessel is on route. On the other hand, if a vessel is at port, the activation of the same threat will not have any observable impact. Utilizing the threat profiling approach while parsing and searching through a series of known sources, ranging from social media to threat and vulnerability catalogs for references of incidents related to specific CAPEC categories or ATT&CK tactics, further information can be extracted to support the threat level calculation and to build threat characteristics that can be compared to threat agent characteristics.

##### Step 2—Threat Agent Mapping

In the second step, threat agents are mapped to each situation. For example, while some threat agents may apply to all situations (e.g., the threat agent ‘cyber-criminal’ may be applied in all the situations defined in Figure 4), other threat agents may only apply to specific situations (e.g., ‘pirates’ are only applicable to situations S6–S8). To fully identify threat agents, we utilize Intel’s Threat Agent Library (TAL) and inherit the profile characteristics for maritime-specific profile instances. This implementation essentially supports an extra layer of filtering, since it allows the risk assessor to not only map the active threats per situation, but also to match them with the threat agents that are expected to have sufficient capabilities to activate threats. We adopt the approach of [31], where threat agent capabilities are expressed as Common Vulnerability Scoring System (CVSS) v3 vectors. The purpose of this exercise is to map threat agents along with the exact attributes that express their capability of exploiting cataloged vulnerabilities. For example, a threat agent that has been attributed a capability of ‘Adjacent Network’ attack vector, is able to to exploit vulnerabilities that require either ‘Network’ or ‘Adjacent Network’ attack vector, but cannot exploit vulnerabilities with ‘Local’ or ‘Physical’ attack vector. For threat assessment, different threat agents may apply in each situation and different threat agents can take advantage of different vulnerabilities based on their capabilities, which subsequently affects the underlying vulnerability map enabled by each threat.

##### Step 3—Situational Threat Likelihood and Profile Filtering

Combining the output of the previous steps an information map is derived, where a set of attributes that resemble ones used to characterize threat agent profiles in known taxonomies is observed, more specifically resources required and skills required from CAPEC can be connected to an attackers capability characteristics found in TAL. At the same time, consequences from the CAPEC catalog and tactics from ATT&CK can be connected to an attackers motivation. Utilizing the cataloged characteristics of both threats and threat agents, we can produce a refined threat likelihood level.

#### 3.2.3. Situational Vulnerability Assessment

Again, as in threat assessment, while in MITIGATE, vulnerability assessment is situation-agnostic, in the proposed methodology, the vulnerabilities are automatically assessed according to each situation, as described below.

##### Step 1—Vulnerability Identification

This step focuses on the identification and assessment of confirmed vulnerabilities of assets, which can be exploited and lead to successful attacks. Our methodology utilizes the National Vulnerability Database (NVD) [32], which contains over 160.000 detailed entries in a structured format, along with other reliable online sources to identify the characteristics of vulnerabilities. Vulnerabilities are product-based, which means that they are targeted towards a specific asset, be it hardware, operating systems, or applications; such components are listed in the Common Platform Enumeration (CPE) catalog, while their connections to Common Vulnerabilities and Exposures (CVE) entries reside in NVD. Therefore, the list of individual vulnerabilities of the assets from the asset modeling step can be created from existing connections between the two catalogs.

##### Step 2—Vulnerability Scoring

Once the vulnerabilities are identified, then it is necessary to determine its exploitability metrics. Our methodology utilizes the CVEs provided by the National Vulnerability Database (NVD), which are recorded along with part of their CVSS v3.1 and v2.0 attributes. The MITIGATE tool utilizes the CVSS v2 exploitability metrics to define a vulnerability level as illustrated in Table 1. By combining the available exploitability characteristics for each recorded vulnerability (i.e., CVE) a single value is produced to express the vulnerability level based on the CVSS score, as defined in [6].

##### Step 3—Vulnerability Score Assessment

Changes in the asset model along with its interconnections from one situation to another may affect the vulnerability level of the assets. As the connectivity of networked systems, as well as their physical and logical accessibility depends on the situation, the vulnerability list produced for the same asset in a different situation may differ. For example, when an asset with a vulnerability that has a ‘Network’ attack vector is not connected to a network with internet connectivity, the vulnerability does not apply in this case and may only be activated by an ‘Adjacent network’ vector.

##### Step 4—Threat Agent Scoring

Threat Agent Capabilities and Vulnerabilities: Since maritime services take place in a vast, dynamic environment, the human actors and more specifically the threat agents active around each service are multiple and may differ. Throughout this step, we utilize real life incidents to place threat agent profiles in the context of specific maritime services. Furthermore, to characterize the threat agents with specific attributes that can directly be compared to specific vulnerabilities, we use the approach from our previous work [31]. In [31], we expressed threat agent profiles from the healthcare sector as CVSSv3 *capability vectors*, which we compared to CVSSv3 vulnerability vectors. We inherit the recorded characteristics for similar profiles in the maritime environment, which presents a final underlying challenge for this step. The vectors need to be translated to CVSSv2 in order to be compatible with the mitigate solution. The transformation of CVSSv3 vectors to CVSSv2 vectors is based on the approach of [33].

#### 3.2.4. Situational Impact Assessment

##### Step 1—Impact Identification

To derive the impact that existing vulnerabilities may cause, the list of vulnerabilities procured by the Situational Vulnerability Assessment is parsed and the impact section of the CVSS vector is extracted to another list. This section contains three values referring to confidentiality integrity and availability impact, which are combined to produce a single value in the next step.

##### Step 2—Impact Level Calculation

This step focuses on the Impact level calculation, which measures the effect that can be expected as the result of the successful exploitation of a vulnerability that resides in a critical asset. In CVSS, the three security criteria Confidentiality (C), Integrity (I) and Availability (A) are rated in a three tier scale of [None,Low,High]. We can define a mapping from the three tier scale onto a five-tier scale ranging from Very Low (VL) to Very High (VH) to combine these three characteristics (see Table 2). This will provide a single estimation for the overall impact of a specific asset/vulnerability combination. As defined in the underlying MITIGATE methodology, the confidentiality, integrity and availability sub-scores of the recorded vulnerabilities are used as input, to output a single impact level.

##### Step 3—Situational Impact

The impact of security attacks may also vary according to the situation. In a typical risk assessment method, impact is based on the ‘worst-case’ scenario. For example if the unavailability of a navigation system has a very high impact when the vessel is on route, the corresponding impact value will be used while assessing all possible threats that may result in the unavailability of the system in all situations. By applying different impact values according to the situation, more fine-grained risk values will be produced according to the situation. The situational impact values are derived by the combination of the initial impact values and the asset criticality set for each asset in the context of a situation, as shown in Table 3.

Essentially the impact level calculated in the previous step is refined based on the asset *criticality per situation* to output a *situational impact* value. Since the same asset may be more important in different situation, a criticality level is defined for each asset per situation. For example the criticality level of a GPS system may be high while the vessel is on route, but may be low when the vessel is on the port. This criticality level is used to weight the initial impact level defined in the previous step.

#### 3.2.5. Situational Risk Assessment

Finally, by combining the situation based asset models, threat, vulnerability and impact assessment results, the risk analysis engine will output the relevant risks, along with their assessed values, for each different situation. Let *A* denote an asset under examination and T a threat identified throughout the Situational Threat Assessment. Then, the situational risk level caused on asset *A* by threat T in situation *S* is defined as shown below:

(1) $$R_{S}\left(A,T\right)=T_{S}\left(A,T\right)⊗V_{S}\left(A\right)⊗I_{S}\left(A\right)$$

In Equation (1), $T_{S}\left(A,T\right)$ represents the threat level calculated throughout the Situational Threat Assessment step, $V_{S}\left(A\right)$ represents the vulnerability level calculated throughout the Situational Vulnerability Assessment step and finally $I_{S}\left(A\right)$ represents the impact calculated throughout the Situational Impact Assessment step, as defined in Section 3.2.2, Section 3.2.3 and Section 3.2.4, respectively. The resulting situational risk level is computed based on the risk table defined in MITIGATE [5], ranging in the scale from Very Low to Very High.

#### 3.2.6. Situational Aware High Level Security Policy

As the risk assessment results are dynamically computed for each situation, granular security policies can be defined for different situations. Instead of producing a static list of security controls, expressed as high level policies, the suggested security controls will be fine-grained based on the different risks that correspond to each situation. The high-level security policy will then be further refined and instantiated to particular security controls in the next phase of the methodology.

##### Step 1—Existing Control Identification and Assessment

This step reviews the identified vulnerabilities and threats from the previous phases and identifies the level of mitigation based on the existing controls. First, the implemented controls per asset per situation are identified and listed. The existing security controls may provide partial or full mitigation of the effect of existing threats or vulnerabilities. A decision making process based on the existing risks is implemented to illustrate the functionality of existing controls.

##### Step 2—Situational Control Identification and Application

Utilizing the information procured by the previous step, the residual levels of threats, vulnerabilities and risks calculated while incorporating the effect of the initial security controls are mapped. Having identified these values, further security controls that will fully mitigate risks can be suggested. To achieve this result, two approaches are considered:

- Applicable controls for techniques cataloged in the (ATT&CK) framework are listed in MITRE’s D3FEnd Matrix.
- Applicable controls for existing vulnerabilities can be found in the NVD’s references for each individual vulnerability.

### 3.3. Phase 3: Situation-Based Policy Deployment

The last phase of our methodology consists in enforcing these situational controls. This involves refining the situations elicited in Phase 1 and the high-level situational security controls produced in Phase 2 into low level rules that can be deployed by a security management system.

A situation is a particular time frame of interest with a beginning, a life span and an end [34]. The beginning and the end of a situation can determined by combining multiple events coming from multiple sensors and occurring at different moments [9]. Indeed the beginning and the end of a situation involving multiple entities and multiple conditions cannot be limited to simple events captured by one single sensor. Moreover, events being instantaneous, combining multiple events requires complex temporal operators (event ordering, event existence/absence, time windows, etc.) to specify the beginning and end of situations. Complex Event Processing (CEP) provides such features. CEP is “*a defined set of tools and techniques for analyzing and controlling the complex series of interrelated events that drive modern distributed information systems*” [35]. CEP solutions allow specifying complex events through complex event patterns that match incoming event notifications on the basis of their content as well as some ordering relationships on them. Thereby, the beginning and end of the situations elicited during the situation elicitation phase are expressed in a CEP language. The specification of the situations depends on the sensors available in the vessel and their characteristics. Different patterns for describing situations using CEP have been proposed in [8,9]. The resulting low level situations specification is then provided to a situation manager that continuously calculate the current situation.

In parallel, situational security controls are refined into situation-based security policies in order to be enforced by a situation-based security decision making entity. In our approach, situations are specified and calculated at the situation manager side. Therefore, the security policy refers to them only. Hence, we represent situation-based security policies in a generic way as: when *situation* and *some condition* then *authorization decision and/or obligation(s)* where the condition statement is any constraint on any characteristic of the entities involved in the situation as well as the situation itself [8]. This generic approach is flexible enough to express changes of security controls when the situation is shifting to another one using the reactive rules pattern: when *situation* and *situation starts* [and *some condition*] then *obligation(s)* where the obligations reflect the security controls modifications the security management system will enforce. Situation-based authorization rule is another pattern for specifying adaptive authorization controls: when *situation* and *some condition* then *authorization decision)*.

Both the low level situation specification and the situation-based security policy are injected into the security management system [8]. The actors of our deployment architecture (Figure 3) are the following:

- The *sensors* produce context events. A sensor can be any system available in the target vessel that can trigger context events, such a physical button activated by a human, an intrusion detection system, an alarm, a GPS, a proximity sensor, etc.
- The *situation manager* continuously calculates situations according to a low level situation specification. It consumes context events triggered by the sensors and produces situation events. A situation event contains the beginning of the new situation and the end of the last active situation.
- The *control center* is the brain of our security deployment framework as it performs the security decision making process. It consumes both context and situation events, takes security decisions based on a situation-based security policy and produces decision events. Multiple control centers can be deployed for scalability and/or performance reasons. Different strategies can be considered to coordinate decisions [36,37].
- The *actuators* only consume decision events and enforce security controls. Actuators can be any system that can be controlled by a software (e.g., a door that can be locked/unlocked, configurable IT systems, etc.)
- The *event broker* is the distribution middleware that transmits all the events between the actors following the publish-subscribe pattern. The broker divides events into three topics: context events, situation events and decision events. The broker also ensures that only authorized actors (sensors, actuators, situation manager and the command center) can access it.

## 4. Case Study—Applying the Proposed Methodology in the Maritime Cargo Transfer Service

To demonstrate and validate the methodology proposed in Section 3, we will apply it in the the maritime cargo transfer service scenario presented in Section 1.

### 4.1. Situations Elicitation in the Maritime Cargo Transfer Service

In our test scenario, situations will be defined, among others, on the location of the vessel (e.g., vessel is on port or at the sea) or on information related with the threat status of a vessel on route (e.g., high risk areas)—see Figure 2. The map can later be utilized to unveil interconnectivities of the systems, since it works as a searchable graph, upon which the user can specify entry points and target systems to view potential attack paths. The elicitation process of our case study resulted in the eight following situations, as defined in Figure 4:

- **Situation S1**—the vessel is on port while loading the cargo.
- **Situation S2**—the vessel is on port while unloading the cargo.
- **Situation S3**—the vessel is at sea in a pretty safe area and alone.
- **Situation S4**—the vessel is at sea in a pretty safe area nearby another vessel which is known (it has identified itself).
- **Situation S5**—the vessel is at sea in a pretty safe area nearby another vessel which is unknown (it has not identified itself).
- **Situation S6**—the vessel is at sea in a dangerous area and alone.
- **Situation S7**—the vessel is at sea in a dangerous area nearby another vessel which is known (it has identified itself).
- **Situation S8**—the vessel is at sea in a dangerous area nearby an unknown vessel (it has not identified itself).

### 4.2. Situation-Based Risk Assessment in the Maritime Cargo Transfer Service

Based on the methodology, we first identify a critical service, that will act as a test scenario. In this case, the cargo transport service is examined. Utilizing the situation elicitation showcased above, distinct asset maps for distinct situations are cataloged.

With this approach, we extend the asset models of existing maritime risk assessment methodologies like MEDUSA [4] and MITIGATE [5,6], to define situational asset models that capture the modifications of the interconnections between assets and services with respect to the set of defined situations. The available asset model per situation is illustrated in Table 4. In different situations, the same asset map might have specific nodes turned off due to costs or security related reasons.

Then for each situation, the corresponding threat agents are identified and assessed; the threat agents identified for each situation derived from the cargo transport service are listed in Table 5. In the same way, system vulnerabilities are again examined for each situation, with respect to the particular situation asset model. Finally, fine-grained impact values are assigned to systems, based on the importance of different systems in each situation and a risk assessment procedure is initiated for each situation.

Browsing the risk assessment results, we handpick and showcase indicative scenarios of attacks, risks and mitigation controls for each situation. The application of our situation-based, adaptive security framework is summarized in Table 6. In the following paragraphs, we analyze the effectiveness of our approach in identifying and enforcing effective and cost efficient security controls.

#### Case 1. Situational Risks during Cargo Loading/Unloading (S1 & S2): Attacking Admin Applications

Here, we examine attack scenarios involving systems that are accessible by admins while the vessel is on the port for loading/unloading. The Admin FTP client is vulnerable to CVE-2008-3734, which allows remote FTP servers to cause a denial of service (application crash) or possibly execute arbitrary code via format string specifiers in a connection greeting (response). Through this vulnerability the configuration of the FTP server can be changed to accept executable files. Furthermore, Adobe Reader has CVE-2011-2440, which allows attackers to execute arbitrary code via unspecified vectors. Finally, the Admin Operating System’s font library has CVE-2016-0145, which allows attackers to execute arbitrary code via a crafted embedded font. An attacker combining and utilizing these vulnerabilities can get initial access to the admin system and then elevate their privileges. In the scenario we built, a Corrupt Port Official could initially craft two malicious manifests and upload them to the FTP Manifest Database. Throughout the Cargo loading the ship is connected to the port’s network for updates; in that time frame the admin ftp client’s port is open to the adjacent network, and therefore vulnerable to arbitrary code execution. Utilizing this vulnerability, a Mobster group that cooperates with the Corrupt Port Official, notifies their Internal Spy to change the ftp configuration through its vulnerability so the ftp client accepts the malicious file. This is a viable attack in the context of the Cargo Loading and Unloading situations. One possible result of such an attack would be to either load dangerous, illegal cargo in the Cargo Loading or to claim such items in the Cargo Unloading. To mitigate the risks produced by these vulnerabilities, security controls are suggested. For the admin ftp client and the admin operating system the initial risk drops from Very High to Medium by employing file hash comparisons to detect known malware since this method partially mitigates the threat. For the Adobe reader, the risk drops from Very High to Very Low, by applying the patch provided by Adobe the vulnerability is fully mitigated.

#### Case 2. Situational Risks at Sea without Vessel Proximity (S3 & S6). Attacks against Admin and SCADA Systems

Another indicative scenario we investigate is the case of attacks against admin systems throughout travels in safe or unsafe sea, but without any physical proximity with other vessels. The Admin Web Browser is vulnerable to CVE-2015-6144, which allows remote attackers to bypass a cross-site scripting (XSS) protection mechanism via unspecified vectors. In this scenario, an overworked disgruntled employee accesses an unsafe URL contained in a phishing email, sent by a disgruntled maritime systems administrator. Through this vulnerability, the attacker gains initial access to the system and utilizes it to enumerate other components. Afterwards, the attacker targets the Admin Wincc SCADA component that resides in the same OS when the vessel reaches an unsafe sea. Admin Wincc SCADA is vulnerable to CVE-2015-0016, which is a Directory traversal vulnerability that allows remote attackers to gain privileges via a crafted pathname in an executable file. Utilizing the initial access and this vulnerability, the attacker targets the asset throughout the situation S6 to take control of SCADA systems throughout the ship and create disarray. The initial risk for the web browser drops from Medium to Very Low by monitoring access to the Admin Web Browser and by applying a strong password policy or dual authentication for web admin access. For the SCADA system, the initial risk drops from Very High to Very Low by removing the TSWbPrxy from the IE Elevation Policy, which completely mitigates the vulnerability.

#### Case 3. Situational Risks at Sea including Vessel Proximity (S4, S5, S7, S8). Attacks against Navigation Systems

The final scenario, we present is the case of attacks against navigation systems throughout travels in the safe and unsafe sea. Throughout situations S3, S4 and S5, the GPS system of the ship is online. The GPS tracker is vulnerable to CVE-2017-5239. Due to a lack of standard encryption when transmitting sensitive information over the internet to a centralized monitoring service, the Eview EV-07S GPS Tracker discloses personally identifying information, such as GPS data and IMEI numbers, to any man-in-the-middle (MitM) listener. This vulnerability can even lead to factory reset of the device which can lead to elevation of privileges. In situation S7, a ‘Nation State actor’ could take advantage of this vulnerability, steal confidential information about previous routes of the vessel and factory reset the device to either elevate their privileges or erase their traces. In situation S5, we identify the threat agent ‘Pirate’, who could utilize the aforementioned vulnerability to discover the route of the vessel and the best available location to physically board it. For each instance, we apply different security controls, proportionate to the level of the risk. While in S3, we apply no controls, but since the threat level is lower, the existing vulnerability produces a medium risk. In situation S4, applying a vendor-supplied patch is suggested to prevent the device from allowing unauthenticated factory resets without physical access to the device drops the initial risk from medium to very low. In situation S5, we propose the utilization of a PKI system to encrypt communications, which completely mitigates the risk, which again drops from Medium to Very Low. The additional communication cost for PKI verification will only be applied under this specific situation. Through situations S7 and S8, instead of using the GPS system, the vessel utilizes a satellite connection and exchanges data through the Inmarsat Amosconnect asset. This asset is vulnerable to CVE-2017-3222, hard-coded credentials in AmosConnect 8 allow remote attackers to gain full administrative privileges, this way attackers gain the ability to execute commands on the Microsoft Windows host platform with SYSTEM privileges, by abusing AmosConnect Task Manager. In S7, a Nation State actor enumerating information about vessels in an unsafe sea has the skills and resources to take advantage of the aforementioned vulnerability in order to enumerate information about the vessel systems, route, etc. In S8, a Cyber Terrorist has the ability to remotely exploit the same vulnerability and shut down the vessel navigation systems in order to create disarray and damages. In S7, we propose the deletion of all hard-coded credentials, which drops the initial risk from very high to low. Finally, in S8, we propose the application of a strong password policy which drops the initial risk from Very High to Very Low completely mitigating the existing vulnerability.

### 4.3. Situation-Based Policy Deployment in the Maritime Cargo Transfer Service

**Figure 6:**
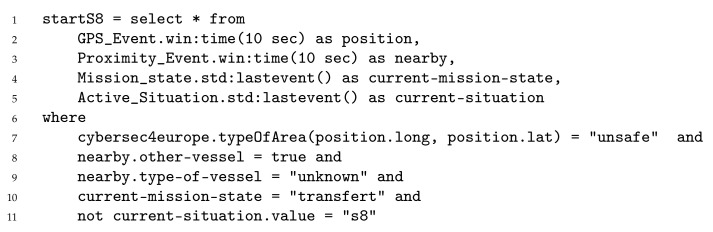
Specification of situation S8 in EPL.

Finally, situations and situational security controls must be refined in order to configure the situation manager and the command center of the dynSMAUG deployment architecture. The situation manager was implemented using Esper, a CEP engine developed by EsperTech (https://www.espertech.com/esper (accessed on 15 November 2021)) under an open source license. Esper includes a language called Event Processing Language (EPL) which is a SQL-standard language with temporal operators extensions (e.g., windows definition and interaction, timed-data arithmetic definition, etc.) Figure 6 depicts the EPL rule that specifies the beginning of situation S8 ‘the vessel is at sea in a dangerous area nearby an unknown vessel’. We hypothesize that the vessel can provide three sensors: a GPS, a proximity sensor that can provide information about nearby vessels, and a vessel workflow sensor that states the current step in the cargo transfer process. Lines 2 and 3 take the GPS and proximity events in a 10 s time frame while lines 4 and 5 retrieve the last mission state and active situation events. Similarly to SQL, the *where* clause allows filtering the request to specific conditions. Here, the position shall be in an unsafe area (line 7), there is a nearby unknown vessel (lines 8 and 9) and the vessel is transferring the cargo (i.e., at sea line 10). When the situation manager detects that situation S8 is starting, it generates a situation event informing that the current situation ends and S8 starts.

**Figure 7:**
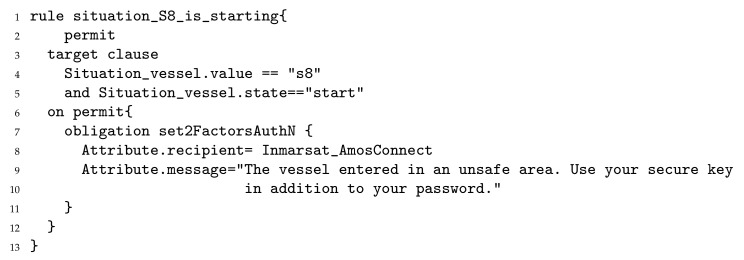
Sample of the low level situation-based security policy.

The command center takes as input situation-based security policies in XACML v3 (eXtensible Access Control Markup Language [38]) or ALFA (Abbreviated Language for Authorization [39]) format. Figure 7 is the ALFA rule that refines the situational security control relating to applying strong password policy in situation S8. We decided to require, when S8 starts (lines 5 and 5), a 2nd factor for authenticating on the Inmarsat AmosConnect (lines 7–9). We consider an actuator system can switch from simple password authentication and two factors authentication. A configurable message can be controlled in the policy (line 9). Two factors authentication negatively impact users experience by making the system more complex to use. Thanks to situational security measures, two factors authentication is only required when needed.

## 5. Discussion and Conclusions

In contrast to existing maritime specific risk assessment frameworks, which are static in terms of security policy enforcement, the proposed framework is dynamic by design. As the security risk of the examined systems is affected by events and situations, the resulting risk level also varies, leading to situation-specific enforcement of security controls. By defining situational asset and threat models, we continuously map active assets to active threat agents for each situation, thus filtering out risks that are not active or very low, in various situations. Following this process, we avoid overbearing the vessel and port systems with security controls targeted towards inactive attack paths. This allows us to avoid a policy of ‘always defaulting in the highest risk’, which in practice may lead to reduced security controls due to lack of resources, lack or efficiency in procedures or other environmental constraints.

In addition, the proposed framework supports automation of the security policy enforcement, by allowing the implementation of automated security policies per situation. By sensing events that indicate changes in the situation such as current location or proximity with known or unknown vessels, it is possible to dynamically adapt the applied security controls to the corresponding situational risk level. The risk assessment phase is semi-automated, as various steps require manual intervention. For example, to introduce further automation to the risk assessment procedure, further research is required in the context of identifying applicable threat agents and groups in specific geographical coordinates, based on past data of recorded attacks.

Another point that needs further work is assessing the level of confidence/assurance of the situation calculus. Indeed, a calculated current situation might deviate from the actual current situation, due to low quality sensors or due to attacks against the sensors themselves. We need to improve our methodology to cover these risks. Interesting approaches related to the concept of *Quality of Context* and *Quality of Situation* [40,41] may provide useful insight towards this direction. Secondly, specifying complex situations using a rule-based language such as EPL may be error prone for very complex situations with many entities or context information. Complementary approaches that may handle this problem may include building up a simulation environment for testing/validating situation specifications and tracking down real environments that can be mapped thoroughly enough to validate them. In addition the application of machine learning techniques could provide initial steps towards the automatic identification of situations in which each asset of an infrastructure resides. Autonomous vessels are challenging environments in the context of such research efforts. Due to the heavy monitoring and control systems already applied to existing autonomous vessels, replicating the asset maps used in the context of a simulation environment could be based on logs exported from monitoring software. At the same time log files that catalog the traffic for each asset could be analyzed with the use of machine learning techniques, to automatically identify applicable situations per asset, based on the recorded events it triggered. We plan to extend our future work towards these directions.

## Figures and Tables

**Table 1:** Vulnerability level calculation matrix.

AV		Local			Adjacent			Network		
	AC	Low	Medium	High	Low	Medium	High	Low	Medium	High
AUTH										
Multiple		VL	VL	L	L	L	M	M	M	H
Single		VL	L	M	L	M	H	M	H	VH
None		L	M	M	M	H	H	H	VH	VH

**Table 2:** Impact level calculation matrix.

C		None			Low			High		
	I	None	Low	High	None	Low	High	None	Low	High
A										
None		VL	VL	L	L	L	M	M	M	H
Low		VL	L	M	L	M	H	M	H	VH
High		L	M	M	M	H	H	H	VH	VH

**Table 3:** Situational impact calculation.

Initial Impact	Asset Criticality		
	Low	Medium	High
Very Low	VL	L	L
Low	L	L	M
Medium	L	M	H
High	L	H	H
Very High	M	H	VH

**Table 4:** Active asset model per situation in the cargo transport service.

	Situations							
Assets	S1 Cargo Loading	S2 Cargo Unloading	S3 Transfer (Safe_Alone)	S4 Transfer (Safe_Prox_K)	S5 Transfer (Safe_Prox_UK)	S6 Transfer (Unsafe_Alone)	S7 Transfer (Unsafe_Prox_K)	S8 Transfer (Unsafe_Prox_UK)
Admin Adobe Reader	X	X	X					
Admin FTP Client	X	X	X					
Admin Operating System	X	X	X	X	X	X	X	X
Admin SSH client			X	X	X	X	X	
Admin Web Browser	X	X	X	X	X	X		
Admin Wincc SCADA			X	X	X	X	X	X
Inmarsat AmosConnect						X	X	X
GPS			X	X	X			
AIS Gateway	X	X						
VTS	X	X						
FTP (Manifest Storage)	X	X						
Web Services	X	X						

**Table 5:** Applicable threat agents per situation for the cargo transport service.

	Situations							
Threat Agents	S1 Cargo Loading	S2 Cargo Unloading	S3 Transfer (Safe_Alone)	S4 Transfer (Safe_Prox_K)	S5 Transfer (Safe_Prox_UK)	S6 Transfer (Unsafe_Alone)	S7 Transfer (Unsafe_Prox_K)	S8 Transfer (Unsafe_Prox_UK)
Disgruntled Employee	X	X	X	X	X	X	X	X
Disgruntled Maritime Systems Administrator (Internal Spy)	X	X	X	X	X	X	X	X
Cyber Criminal Group (Mobster)	X	X		X	X		X	X
Cyber Terrorist	X	X	X	X	X	X		
Nation State	X	X		X	X		X	X
Pirate					X			X
Corrupt Port Official	X	X						

**Table 6:** Final results.

Situations	Situational Risk Assessment (Indicative Risks per Situation)						Situational Risk Mitigation (Relevant Security Controls)		
	Asset	Threat Agent	Threat	Vulnerability/ Vuln. Level	Impact Level	Risk Level	High Level Security Control (DEFEND)	Specific Mitigation	Risk after Mitigation
**S1 & S2:** Cargo Loading & Unloading	Admin Adobe Reader	Corrupt Port Official	CAPEC - 10	CVE-2011-2440: VH	VH	VH	Software Update	Patch Software.	M
	Admin Operating System	Corrupt Port Official	CAPEC - 100	CVE-2016-0145: VH	VH	VH	File Hashing	Employing file hash comparisons to detect known malware.	M
	Admin FTP client	Internal Spy	CAPEC - 137	CVE-2008-3734: VH	VH	VH	File Hashing	Employing file hash comparisons to detect known malware.	M
**S3:** Transfer Safe_Alone	Admin Web Browser	Disgruntled Employee	CAPEC - 588	CVE-2015-6144: VH	VL	M	(1) Resource access pattern analysis (2) Strong password policy	(1) Monitor access to Admin web access (2) Apply strong password policy or dual authentication for web admin access.	VL
	GPS	Nation State	CAPEC - 628	CVE-2017-5239: VH	L	M	-	-	L
**S4:** Transfer Safe_Prox_K	GPS	Nation State	CAPEC - 628	CVE-2017-5239: VH	L	M	Software Update	Applying a vendor-supplied patch to prevent the device from allowing unauthenticated factory reset without having physical access to the device.	VL
**S5:** Transfer Safe_Prox_UK	GPS	Pirate	CAPEC - 628	CVE-2017-5239: VH	L	M	Message Encryption	Utilize the PKI system to encrypt communications	VL
**S6:** Transfer Unsafe_Alone	Admin Wincc SCADA	Disgruntled Maritime Systems Administrator	CAPEC - 76	CVE-2015-0016: VH	VH	VH	Mandatory Access Control	Remove TSWbPrxy from the IE Elevation Policy	VL
**S7:** Transfer Unsafe_Prox_K	Inmarsat AmosConnect	Nation State	CAPEC - 167	CVE-2017-3222: VH	VH	VH	Strong Password Policy	Delete all hard-coded credentials.	VL
**S8:** Transfer Unsafe_Prox_UK	Inmarsat AmosConnect	Cyber Terrorist	CAPEC - 167	CVE-2017-3222: VH	VH	VH	Strong Password Policy	Apply a strong password policy.	L
